# The detection of delta-9-tetrahydrocannabinol, delta-8-tetrahydrocannabinol, delta-10-tetrahydrocannabinol, and cannabidiol in hair specimens

**DOI:** 10.1093/jat/bkaf079

**Published:** 2025-08-19

**Authors:** Amy Racines, Joseph Jones, Katie Lea, Donna Coy

**Affiliations:** United States Drug Testing Laboratories, LLC, Des Plaines, IL 60018, United States; United States Drug Testing Laboratories, LLC, Des Plaines, IL 60018, United States; United States Drug Testing Laboratories, LLC, Des Plaines, IL 60018, United States; United States Drug Testing Laboratories, LLC, Des Plaines, IL 60018, United States

**Keywords:** hair analysis, delta-8-tetrahydrocannabinol, delta-9-tetrahydrocannabinol, delta-10-tetrahydrocannabinol, cannabidiol

## Abstract

Cannabinoid use and misuse has been rising since 2011, and the development of new cannabinoid derivatives, partially due to the passage of the Farm Bill in 2018, and more relaxed legislation, has complicated testing for this drug class. The impact on child welfare in homes with cannabis substance use remains a concern, so detection of environmental exposure to cannabinoids is hugely beneficial. This article reports a validated confirmation method which detects delta-9-tetrahydrocannabinol (Δ9-THC), delta-8-tetrahydrocannabinol (Δ8-THC), delta-10-tetrahydrocannabinol (Δ10-THC), and cannabidiol (CBD) in environmentally exposed hair specimens via liquid chromatography tandem mass spectrometry (LC-MS-MS) following a supported liquid extraction. From February 2024 to October 2024, 30.5% (*n* = 1787) of specimens tested positive for at least one analyte. The most common analyte was Δ9-THC (26.8%, *n* = 1574), followed by Δ8-THC (9.0%, *n* = 528), CBD (6.1%, *n* = 359) and Δ10-THC (0.4%, *n* = 24). While most of the specimens contained multiple analytes, it was found that 21.4% of the positive specimens had a single analyte exposure: 1062 specimens only confirmed for Δ9-THC, 165 specimens only confirmed for Δ8-THC, and 28 specimens only confirmed CBD. The addition of Δ8-THC, Δ10-THC, and CBD to the cannabinoids assay improved the detection of cannabinoids related cases, increasing our total positivity rate by an additional 3.6% (*n* = 213). The detection of all these analytes is crucial for reliable and accurate detection of cannabinoid environmental exposure.

## Introduction

Cannabis use continues to be a prevalent public health concern being the most used illicit drug according to the 2023 National Survey on Drug Use and Health [1]. The major psychoactive ingredient, delta-9-tetrahydrocannabinol (Δ9-THC), is widely tested in a variety of matrices for forensic toxicology testing. According to the National Conference of State Legislatures, twenty-four states, including Washington DC, have legalized recreational use of cannabis [2]. However, testing remains valuable in environments such as workplace drug testing, driving under the influence, and child protection cases.

Since Δ9-THC remains classified as a Schedule I drug by the Drug Enforcement Administration, synthetic cannabinoids have been developed to circumvent regulations. The passage of the Farm Bill in 2018 legalized hemp, further complicating cannabis testing [3]. Hemp-derived cannabinoids, like delta-8-tetrahydrocannabinol (Δ8-THC) and delta-10-tetrahydrocannabinol (Δ10-THC) could now be synthesized from cannabidiol (CBD), creating additional challenges in the detection of cannabinoids through established drug testing methods where only Δ9-THC and/or metabolites were analyzed.

Accurate detection and identification of children in homes with cannabis use is crucial for the children’s well-being. The relaxation of state laws and legalization of hemp surrounding marijuana has been linked with a decrease in the perceived risk of marijuana [4]. However, the impact on child welfare in homes with cannabis substance use remains a concern. Parents who use marijuana are three times more likely to engage in physical abuse of their children, specifically boys and older children [5]. This increase in physical child abuse is also seen in regions with greater density of cannabinoid dispensaries [5].

In addition to the potential physical danger, children in homes with cannabis substance use can also exhibit medical problems. These children are at risk for marijuana poisonings, as increased poisonings of children under 12 years have been linked to medical marijuana use in Colorado [6]. Secondhand exposure of children to marijuana in homes with cannabis use is yet another concern, as children have produced positive urine tests from secondhand exposure in their homes [7]. Secondhand exposure to children can also lead to respiratory infections [8]. Identifying environmental exposure to substances in children provides an avenue of evidence-based testing that can be beneficial in child protection cases. One non-invasive approach is hair testing children in environments where substance use is suspected.

In this study, the aim was to use hair drug testing to detect environmental exposure of Δ8-THC, Δ9-THC, Δ10-THC, and CBD and report their prevalence in a high-risk population. Hair analysis methodology included initial testing by enzyme-linked immunosorbent assay (ELISA) and confirmation by liquid chromatography tandem mass spectrometry (LC-MS-MS).

## Materials and methods

### Materials

Certified reference materials were purchased from Cerilliant (Round Rock, TX, USA). HPLC grade methanol (MeOH), HPLC grade acetonitrile (ACN), and optima LC-MS formic acid were acquired from Fisher Chemical (Fair Lawn, NJ, USA). HPLC grade methyl tert-butyl ether (MTBE) was acquired from Alfa Aesar (Ward Hill, MA, USA). Type II deionized (DI) water was supplied by a PURELAB Chorus 1 system (Elga LabWater, Woodridge, IL, USA). All calibrators and controls were prepared by fortifying drug-free keratin powder (Spectrum Chemical, Gardena, CA, USA).

### Specimens

A secondary analysis was performed on de-identified hair specimens that had been submitted for routine environmental exposure analysis from 19 February 2024 to 31 October 2024. IRB approval was not required for this project. Hair specimens were trimmed to 1.5 inches from the root to represent approximately 3 months of growth. The purpose of this analysis was to detect environmental exposure, so a wash step was omitted from the procedure.

### Methods

#### Initial testing

Using an OMNI Bead Ruptor Elite (OMNI International, Kennesaw, GA, USA), aliquots (16–24 mg) were homogenized with 5–6 2.3 mm steel ball bearings for 1–3 minutes, or until powdered. MeOH (1.5 ml) was added to each specimen before sonicating for 2 hours at 50°C. The specimens were centrifuged for 5 min at 1500 g, and 1 ml of the supernatant was transferred to a silanized glass tube without disturbing the pellet. After evaporation to dryness under nitrogen at 40°C, 400 µl of drug-free reconstitution buffer (Immunalysis Corporation, Pomona, CA, USA) was added to each specimen with vortex mixing. Extracts were then analyzed using a THC Forensic ELISA kit using manufacturer instructions (Neogen, Lansing, MI, USA) with a cutoff of 1 pg/mg.

#### Confirmatory testing

A second aliquot (16–24 mg) was prepared for confirmatory testing of presumptive positive specimens. Aliquots were homogenized as described above and were sonicated in a warm water bath at 50°C for 2 hours. After centrifugation for 10 mins at 1500 g, the supernatants were filtered through a fitted polypropylene reservoir into 13x100 mm silanized glass tubes, evaporated to dryness under nitrogen at 40°C, and reconstituted in 300 µl of 70:30 MeOH: DI water. The reconstituted residues were purified using an automated supported liquid extraction (SLE) using a Biotage^®^ Extrahera™ Classic instrument (Uppsala, Sweden). The reconstituted specimens were applied to Biotage^®^ Isolute^®^ SLE+ extraction cartridges, and cannabinoids were eluted twice with 600 µl of MTBE. Eluates were evaporated to dryness under nitrogen at 40°C, reconstituted in 100 µl of 60:40 DI water: ACN, and transferred to 2 ml autosampler vials with glass inserts.

Using an Agilent Technology 1200 HPLC system (Wilmington, DE, USA), cannabinoids were chromatographically separated along a Cortecs UPLC C18+ column (Waters, Milford, MA, USA). The solvent system was isocratic, consisting of 45% mobile phase A (95:5 DI water: ACN with 0.1% formic acid) and 55% mobile phase B (ACN with 0.1% formic acid) with a 500 µl/min flow. After 7.5 minutes, the column was flushed with 100% mobile phase B for 1 minute before re-equilibrating the column to the starting conditions for 1 minute. Δ8-THC, Δ9-THC, Δ10-THC, and CBD were detected using a Sciex Triple Quad™ 5500 tandem mass spectrometer using electrospray ionization in positive mode (Foster City, CA, USA). The ion spray voltage was 2500 V, and the source temperature was 650°C. Mass transitions and the corresponding instrument parameters are shown in Table 1. The cutoff concentration of each analyte was 40 pg/mg.

**Table 1.** Mass spectrometry parameters and retention times

Compound	Retention time (min)	Precursor Ion (m/z)	Product Ion (m/z)	Dwell time (msec)	Declustering Potential (V)	Collision energy (V)	Collision cell exit potential (V)
CBD 1	2.2	315.0	193	150	111	31	16
CBD 2	2.2	315.0	123	150	86	45	6
CBD-*d3* 1	2.2	318.0	196	150	38	22	15
CBD-*d3* 2	2.2	318.0	123	150	176	30	15
Δ9-THC 1	5.5	315.1	193	150	141	31	20
Δ9-THC 2	5.5	315.1	123	150	66	45	14
Δ9-THC-*d3* 1	5.5	318.0	196	150	52	22	15
Δ9-THC-*d3* 2	5.5	318.0	123	150	176	30	18
Δ8-THC 1	6.0	315.1	193	150	26	33	10
Δ8-THC 2	6.0	315.1	123	150	71	45	10
Δ10-THC 1	7.6	315.1	193	150	96	33	16
Δ10-THC 2	7.6	315.1	123	150	101	51	14

#### Method validation

The confirmation method was validated based on the American Academy of Forensic Sciences Standards Board standard 036 (ASB 036) [9]. The linear range was determined by five calibration curves over five different runs. Matrix effect was analyzed at 20 pg/mg and 320 pg/mg. The limit of detection and limit of quantitation were calculated using nine different negative lots extracted over three different days. Intra- and inter-day precision was calculated at 20, 50, and 320 pg/mg over five different days. Carryover and crosstalk were analyzed at 20x the cutoff concentration. Extract stability was tested at 24 hours, 48 hours, and 7 days. Ten authentic specimens were analyzed for endogenous interferences. To determine any common exogenous interferences, 41 compounds were evaluated (Table 2).

**Table 2.** The compounds analyzed for interferences in the method

Classification of compound	Interference tested
Cannabinoids	Cannabichromene, exo-tetrahydrocannabinol, cannabidivarin, tetrahydrocannabivarin, cannabicyclol, cannabinol, cannabigerol
Over the counter medications	Ibuprofen, naproxen, ketoprofen, lidocaine, ephedrine, pseudoephedrine, phentermine, dihydrocodeine, phenylpropanolamine, dextromethorphan
Amphetamines	Amphetamine, methamphetamine, 3,4-methylenedioxyamphetamine, 3,4-methylenedioxymethamphetamine, 3,4-methylenedioxy-N-ethylamphetamine,
Benzodiazepines	Oxazepam, temazepam, nordiazepam, lorazepam, 2-hydroxyethylflurazepam, 7-aminoflunitrazepam, 7-aminoclonazepam, 7-aminonitrazepam, α-hydroxyalprazolam, α-hydroxymidazolam, α-hydroxytriazolam
Cocaines	Benzoylecgonine
Opioid	Codeine, morphine, hydrocodone, hydromorphone, oxycodone, oxymorphone
Hallucinogen	Phencyclidine
Other	Ketoprofen, phentermine

## Results

### Method validation

All confirmation validation results satisfied the ASB 036. The linear range of each analyte was 16–400 pg/mg. The limits of detection were 4.8 pg/mg for Δ9-THC, Δ10-THC, and CBD, and 6.4 pg/mg for Δ8-THC. The limit of quantitation was 16 pg/mg for all analytes. Bias and intra- and inter-day precision studies met all acceptance criteria. No carryover was observed at 100X the cutoff, and no crosstalk was observed at 50X the cutoff. Extracts were determined to be stable for up to 7 days. Interference studies determined there were no common endogenous interferences for this assay, but other cannabinoids have some potential to cause interferences. Exo-THC elutes immediately before Δ9-THC, and so very high levels of exo-THC may not be resolved from Δ9-THC, preventing accurate quantitation of Δ9-THC. Very high levels of cannabigerol may interfere with CBD-*d3*, and therefore may prevent accurate reporting of CBD.

### Prevalence data

Between 19 February 2024 and 31 October 2024, 5863 hair specimens were received for routine analysis of environmental cannabinoids exposure. In this sample set, 1787 (30.5%) of those specimens confirmed positive for Δ9-THC, Δ8-THC, Δ10-THC, and/or CBD. Of those specimens, 1574 (88.1%) confirmed positive for Δ9-THC (median = 181.5 pg/mg, interquartile ranges (IQR)=88.0 pg/mg, 488.0 pg/mg), 528 (29.5%) confirmed positive for Δ8-THC (median = 110.0 pg/mg, IQR= 63.0 pg/mg, 244.8 pg/mg), 24 (1.3%) confirmed positive for Δ10-THC (median = 69.5 pg/mg, IQR= 50.8 pg/mg, 90.0 pg/mg), and 359 (21.1%) confirmed positive for CBD (median = 99.0 pg/mg, IQR= 58.5 pg/mg, 218.5 pg/mg).

**Figure 1.**
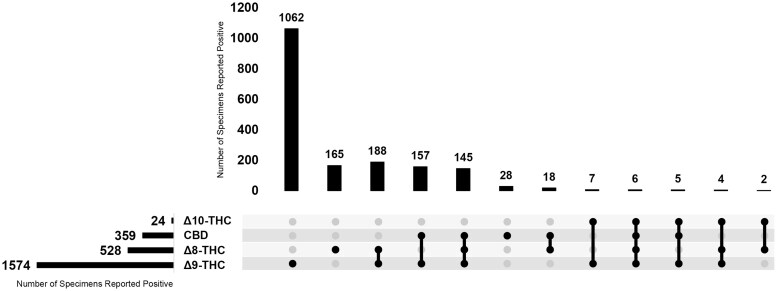
Total Positivity and co-exposure data for Δ9-THC, Δ8-THC, Δ10-THC, and CBD.

Single analyte specimens accounted for 70.4% of the positive specimens, where 1062 (59.7%) of the positive specimens only confirmed for Δ9-THC, 165 (9.2%) of the positive specimens only confirmed for Δ8-THC, and 28 (1.5%) of the positive specimens only confirmed for CBD. There were no specimens that tested positive for Δ10-THC only. The remainder of this population tested positive for multiple analytes (Fig. 1).

## Discussion

This study has presented here a fully validated method using hair for the detection of Δ9-THC, Δ8-THC, Δ10-THC, and CBD to identify individuals exposed to cannabis contaminated environments. A positive cannabinoid rate of approximately 30.5% was reported, which far exceeds normal demographic populations, such as workplace testing [10]. The specimens received at the laboratory are from high-risk populations, such as family courts or substance use disorder treatment centers intake and monitoring. Garcia-Caballero and Martinez-Gonzalez reported that 71% of children suspected to be exposed to abused drugs were positive for cannabinoids [11]. Franz et al noted that cannabinoids were the most frequent substance found (28.9%; *n* = 387) in the hair of children in ongoing child welfare investigations [12].

In this study, Δ9-THC had the highest positivity rate (26.8%), followed by Δ8-THC (9.0%), CBD (6.1%), and finally Δ10-THC (0.4%). In 2022, 20% of U.S. adults reported past year CBD or hemp-derived product use [13]. Similarly, in 2023, 26.3% of survey participants reported past-year cannabinoid usage, 21.1% reported past-year usage of CBD, and 11.9% reported past-year usage of Δ8-THC [14]. These reported statistics are consistent with this reported Δ9-THC and Δ8-THC positivity rate, but higher than what is seen in this data set for CBD. This discrepancy can possibly be explained due to the low cross-reactivity of this study’s screening assay to CBD, as well as the growing popularity of CBD usage. The addition of Δ8-THC, Δ10-THC, and CBD to this cannabinoids assay improved the detection of cannabinoids related cases an additional 3.6% (*n* = 213).

This is the first report of the detection of Δ8-THC and Δ10-THC in hair specimens. Currently, nationally representative prevalence studies of Δ8-THC are limited, and prevalence studies of Δ10-THC and CBD are lacking. Figure 1 depicts the positive results of the population and the various co-exposure combinations, allowing for further statistical calculations on the co-exposure data. Approximately one in five specimens that tested positive for Δ9-THC had co-exposure with Δ8-THC. These findings were higher than a recent survey which noted that 17% of past-month cannabis users self-reported past-month Δ8-THC use [3]. This difference may be explained by the interest in Δ8-THC has increased and continues to amplify following the passage of the Farm Bill in 2018 [3].

Additionally, in this study, one in five specimens that tested positive for Δ9-THC had co-exposure with CBD. LoParco et al reported that 21.1% of past year cannabis users self-reported CBD use, which is consistent with this study’s Δ9-THC co-exposure [3]. Lastly in this data set, about one in two specimens that tested positive for CBD had co-exposure for Δ8-THC, and 87% of specimens that tested positive for CBD had co-exposure for Δ9-THC. These findings are higher than the Drug and Alcohol Dependence Reports’ data from 2022 which indicated almost 2/3 of self-reported CBD users also self-reported using cannabis [13].

The concentrations of Δ9-THC in hair specimens for this study were consistent with other published findings of similar demographics. A single case report showed an estimated 300 pg/mg of Δ9-THC using a semiquantitative immunoassay technique of a child living in a household where both parents were active marijuana users [15]. Moosmann et al reported Δ9-THC concentrations in all but one specimen (*N* = 41) of hair from children living with adult cannabis smokers ranging from 0–1200 pg/mg (mean = 108 pg/mg; SD = 202 pg/mg; median = 35 pg/mg) [16]. Another study of 141 families involving ongoing social services intervention found 23 children between the ages of 7 and 14 with Δ9-THC concentrations ranging from 22 pg/mg to 290 pg/mg [17].

This study has several strengths and weaknesses that should be noted. A strength of this study was the large number of authentic specimens from high-risk cases across the nation. A second strength of the study was the ability to use a readily available immunoassay kit, standard laboratory equipment, and routine LC-MS-MS procedures. A weakness of the study was a lack of access to the case management and/or medical records of the donors. Another weakness of the study was the low cross-reactivity of our initial testing ELISA. According to the ELISA kit product specifications, target compounds Δ8-THC, Δ9-THC, and CBD have cross-reactivities of 5%, 4%, and 0.01%, respectively. Due to the low ELISA cross-­reactivity, the screening cutoff, which targets the metabolite carboxy-delta-9-THC, was set lower than the confirmation cutoff. Another limitation was that it could not be determined if the various cannabinoids detected were incorporated into the hair simultaneously or during different episodes of consumption or exposure. Additionally, in this method, it cannot be determined if the drugs were incorporated into the hair through ingestion or through exposure. Another limitation of this study was that the specimens analyzed were a convenience sampling of specimens forwarded to the laboratory for routine forensic analysis and not obtained from a randomly controlled trial. For obvious reasons, a randomly controlled trial for this type of study is not ethically feasible.

Future directions of this research include an evaluation of co-exposure to other substances of abuse when these target compounds are detected as well as prospective studies that evaluate the long-term outcomes of children identified in these environments. Additionally, future studies can compare this unwashed hair data set to data of hair that is washed to help distinguish ingestion vs exposure.

## Conclusion

This study has presented a fully validated method using hair specimens for the detection of Δ9-THC, Δ8-THC, Δ10-THC, and CBD to identify individuals exposed to cannabis contaminated environments. Due to the negative impact of child welfare in cannabis substance use homes, it is crucial to provide evidence-based testing for these cases. The detection of Δ9-THC alone is not sufficient for environmental exposure identification; other cannabinoids are imperative to produce the most accurate and reliable results.

## Data Availability

The data underlying this article will be shared on reasonable request to the corresponding author. Casework data cannot be shared for privacy reasons.
